# Annexin A8 can serve as potential prognostic biomarker and therapeutic target for ovarian cancer: based on the comprehensive analysis of Annexins

**DOI:** 10.1186/s12967-019-2023-z

**Published:** 2019-09-02

**Authors:** Rui Gou, Liancheng Zhu, Mingjun Zheng, Qian Guo, Yuexin Hu, Xiao Li, Juanjuan Liu, Bei Lin

**Affiliations:** 1 Department of Obstetrics and Gynaecology, Shengjing Hospital Affiliated to China Medical University, Shenyang, 110004 Liaoning China; 2 Key Laboratory of Maternal-Fetal Medicine of Liaoning Province, Key Laboratory of Obstetrics and Gynecology of Higher Education of Liaoning Province, Liaoning, China

**Keywords:** ANXA8, Ovarian cancer, Prognostic value, Annexins, Bioinformatics analyses

## Abstract

**Background:**

Annexins are involved in vesicle trafficking, cell proliferation and apoptosis, but their functional mechanisms in ovarian cancer remain unclear. In this study, we analyzed Annexins in ovarian cancer using different databases and selected Annexin A8 (ANXA8), which showed the greatest prognostic value, for subsequent validation in immunohistochemical (IHC) assays.

**Methods:**

The mRNA expression levels, genetic variations, prognostic values and gene–gene interaction network of Annexins in ovarian cancer were analyzed using the Oncomine, Gene Expression Profiling Interactive Analysis (GEPIA), cBioPortal, Kaplan–Meier plotter and GeneMANIA database. ANXA8 was selected for analyzing the biological functions and pathways of its co-expressed genes, and its correlation with immune system responses via the Database for Annotation, Visualization, and Integrated Discovery (DAVID) and the TISIDB database, respectively. We validated the expression of ANXA8 in ovarian cancer via IHC assays and analyzed its correlation with clinicopathological parameters and prognosis.

**Results:**

*ANXA2/3/8/11* mRNA expression levels were significantly upregulated in ovarian cancer, and *ANXA5/6/7* mRNA expression levels were significantly downregulated. Prognostic analysis suggested that significant correlations occurred between *ANXA2/4/8/9* mRNA upregulation and poor overall survival, and between *ANXA8/9/11* mRNA upregulation and poor progression-free survival in patients with ovarian serous tumors. Taken together, results suggested that *ANXA8* was most closely associated with ovarian cancer tumorigenesis and progression. Further analyses indicated that *ANXA8* may be involved in cell migration, cell adhesion, and vasculature development, as well as in the regulation of PI3K-Akt, focal adhesion, and proteoglycans. Additionally, *ANXA8* expression was significantly correlated with lymphocytes and immunomodulators. The IHC results showed that ANXA8 expression was higher in the malignant tumor group than in the borderline and benign tumor groups and normal ovary group, and high ANXA8 expression was an independent risk factor for survival and prognosis of ovarian cancer patients (*P* = 0.013).

**Conclusions:**

Members of the Annexin family display varying degrees of abnormal expressions in ovarian cancer. ANXA8 was significantly highly expressed in ovarian cancer, and high ANXA8 expression was significantly correlated with poor prognosis. Therefore, ANXA8 is a high candidate as a novel biomarker and therapeutic target for ovarian cancer.

## Background

Ovarian cancer is one of the most common malignant tumors in the female reproductive system. The GLOBOCAN 2018 estimates indicated that ovarian cancer causes approximately 180,000 deaths worldwide each year, with the greatest mortality rate among malignant gynecological tumors [1]. Over 70% of ovarian cancer patients are diagnosed at late stages due to the absence of early typical clinical symptoms and effective diagnostic methods [2]. Despite gradual improvements in surgery and targeted therapeutic drugs, these therapeutic methods still fail to yield a desirable progression-free survival (PFS) in patients with ovarian cancer, and the subsequent treatment of recurrent ovarian cancer still faces challenges [3]. Hence, it has become a current research trend to investigate the mechanisms underlying the tumorigenesis and progression of ovarian cancer and to search for tumor biomarkers with high sensitivity and specificity.

Annexins comprise a Ca^2+^-dependent, phospholipid-binding protein superfamily of 12 members (A1–A11, and A13) that share a conserved core domain containing a Ca^2+^-binding site. In eukaryotic cells, Annexins are mainly involved in various biological activities associated with membrane transport and membrane surfaces, such as vesicle trafficking, signal transduction, cell proliferation, cell differentiation, and apoptosis [4]. Recent findings have revealed abnormal expression levels of Annexins in tumors and their involvement in various biological processes, such as the tumorigenesis and progression, as well as chemoresistance in tumors [5]. Recent studies have shown that ANXA2 is highly expressed in ovarian cancer [6] and that ANXA3 and ANXA4 are involved in cisplatin resistance in ovarian cancer [7, 8]. However, the roles and mechanisms of most Annexins remain unclear. ANXA8 is a member of the Annexin superfamily and was first discovered in complementary DNA libraries generated from human placentas [9]. Previous studies confirmed that ANXA8 is upregulated in numerous types of malignant tumors, such as acute promyelocytic leukemia [10], cholangiocarcinoma [11], breast cancer [12], and pancreatic cancer [13], but its relevant mechanisms have been rarely reported, and its roles in ovarian cancer have not yet been elucidated. We conducted a preliminary screen for differentially expressed genes in three ovarian cancer cell lines and found that *ANXA8* was upregulated in cell lines with greater malignancy and drug resistance [14].

Here, we searched for mRNA expression levels of Annexins between ovarian cancer and normal ovarian tissues using the Oncomine and Gene Expression Profiling Interactive Analysis (GEPIA) databases, analyzed the prognostic value of each Annexin family member in ovarian cancer using the Kaplan–Meier plotter database, and constructed a gene–gene interaction network for Annexins in order to explore their mechanisms of function. We further analyzed ANXA8, which was significantly correlated with the prognosis of patients with ovarian serous tumors. *ANXA8* was subjected to gene set enrichment analysis (GSEA) using The Cancer Genome Atlas (TCGA) database to explore its biological functions and relevant pathways. Correlation between *ANXA8* and the immune system were analyzed using the TISIDB database. In addition, ANXA8 expression in ovarian cancer was evaluated and validated using clinical samples. Our study was aimed at exploring the clinical significance of ANXA8 and providing a theoretical basis for the early diagnosis, prognostic judgments, and targeted therapy of ovarian cancer.

## Methods

### Oncomine analysis

The Oncomine database (http://www.oncomine.org) is an online microarray database that includes 715 datasets, as well as 86,733 cancer and normal tissue samples [15]. In this study, the Oncomine database was employed to analyze the mRNA expression levels of Annexins in different types of cancer. The search was carried out based on the following criteria: (a) type of analysis: cancer versus normal tissues; (b) type of data: mRNA; (c) thresholds: fold change = 2 and *P* value = 0.01.

### GEPIA dataset analysis

GEPIA (http://gepia.cancer-pku.cn/) is a database of data retrieved from the UCSC Xena server, which includes 9736 tumor samples and 8587 normal samples [16]. The database can be used to analyze differential gene expression levels in tumor tissues and paracancerous tissues, as well as patient survival and prognosis. In this study, we validated the differential mRNA expression levels of Annexins in cancerous and paracancerous tissues of ovarian cancer using the database. *P* < 0.05 indicated statistically significant differences.

### TCGA and cBioPortal analyses

The open-source cBioPortal database (http://www.cbioportal.org) contains data retrieved from the TCGA database for interactively exploring cancer genomic datasets [17]. It contains a wide variety of data, including DNA copy numbers, DNA methylation, mRNA and microRNA expression levels, and nonsynonymous mutations. In this study, the cBioPortal database was used to analyze genetic variations in Annexin genes (such as amplifications, deep deletions, and mutations). In addition, the database was used to evaluate correlations between Annexins.

### Kaplan–Meier plotter analysis

As a tool for assessing biomarkers, the Kaplan–Meier plotter (http://kmplot.com) can be used to assess the functions of 54,675 genes and 10,188 tumor tissue samples, including breast cancer, ovarian cancer, lung cancer, and gastric cancer [18]. In this study, we analyzed the prognostic value of Annexins in ovarian cancer using the Kaplan–Meier plotter. The prognostic values of high- and low-expression groups were evaluated according to the hazard ratio (HR), 95% confidence interval (CI), and log-rank *P*-values. *P* < 0.05 indicated statistically significant differences.

### GeneMANIA analysis

GeneMANIA (http://www.genemania.org) provides a flexible web interface for deriving hypotheses based on gene functions [19]. GeneMANIA generates a list of genes with similar functions to the query gene and constructs an interactive functional-association network to illustrate relationships between genes and datasets. In this study, the database was adopted to construct a gene–gene interaction network for Annexins in terms of physical interactions, co-expression, predictions, co-localization, and genetic interaction, as well as to evaluate their functions.

### Functional and pathway enrichment analysis

We identified genes co-expressed with *ANXA8* using the cBioPortal database. A Spearman’s correlation coefficient exceeding 0.30 indicated a good correlation between *ANXA8* and a co-expressed gene. The DAVID database (https://david.ncifcrf.gov) integrates both biological data and analytical tools to provide systematic and comprehensive annotations of biological functions [20]. We employed the DAVID database for functional and pathway enrichment analysis of genes co-expressed with *ANXA8*. *P* < 0.05 indicated statistically significant differences.

### TISIDB analysis

The TISIDB database (http://cis.hku.hk/TISIDB) integrates 988 reported immune-related anti-tumor genes, high-throughput screening techniques, molecular profiling, and paracancerous multi-omics data, as well as various resources for immunological data retrieved from seven public databases [21]. The database enables analyses of correlations for selected genes with lymphocytes, immunomodulators, and chemokines. In this study, we employed the TISIDB database to analyze the relationship between the expression levels of Annexins and clinical stages in ovarian cancer, as well as to investigate correlations between *ANXA8* expression and lymphocytes and immunomodulators.

### Sample sources and clinical data

A total of 122 ovarian tissues were obtained from Shengjing Hospital of China Medical University from 2008 to 2012, and were paraffin-embedded for use. Written informed consent was obtained from all participants. This study was approved by the Ethics Committee of China Medical University. No patients received radiotherapy, chemotherapy, or hormone therapy prior to surgery, and complete clinical data was obtained for each patient. All pathological sections were assessed by pathologists and yielded a clear diagnosis. The patients were divided into four groups, including epithelial ovarian cancer (malignant tumor group, n = 81), epithelial ovarian borderline tumors (borderline tumor group, n = 17), epithelial ovarian benign tumors (benign tumor group, n = 13), or normal ovarian tissues (normal ovary group, n = 11). The median ages of patients in the malignant tumor group, borderline tumor group, benign tumor group, and normal ovary group were 52 years old (36–79 years old), 43 years old (26–79 years old), 50 years old (28–70 years old), and 44 years old (32–65 years old), respectively. No significant difference was observed in age between these groups (*P* > 0.05). In the malignant tumor group, there were 15, 23, and 43 cases with well, moderately, and poorly differentiated tumors, respectively. Based the criteria established by the International Federation of Obstetrics and Gynecology (FIGO 2009), 35 patients had stage I–II ovarian cancer and 46 patients had stage III–IV ovarian cancer. In addition, 19 patients had pelvic and/or para-aortic lymph node metastasis.

### IHC assay

Ovarian tissue paraffin blocks were processed into 5-μm-thick sections. ANXA8 expression was detected using a streptavidin–peroxidase (SP) method. Colon tissue slices showing ANXA8 expression were used as positive controls, phosphate-buffered saline was used instead of the antibody as a negative control, and each batch of slices was analyzed in parallel with positive and negative control slices. Polyclonal antibody against ANXA8 (Abcam, Cambridge, UK; 1:75) was used to evaluate the expression and clinical significance of ANXA8 in ovarian cancer. Staining steps were performed using SP kit. The presence of strong granular staining in the cell membrane and cytoplasm was deemed to be ANXA8-positive. Stained cells were classified based on their color intensity using the following score system: not pigmented (0 score), light yellow (1 score), brownish yellow (2 scores), and dark brown (3 scores). The percentage of stained cells within the microscope field of view was classified as follows: < 5% (0 score), 5%–25% (1 score), 26%–50% (2 scores), 51%–75% (3 scores), and > 75% (4 scores). Multiplying the score of the stained cells and the percentage of stained cells yielded the following final scores: 0–2 scores (−), 3–4 scores (+), 5–8 scores (++), and 9–12 scores (+++). 3–12 scores were considered as positive expression, and 5–12 scores were considered as high positive expression. Each tissue section was assessed independently by two observers to reduce errors.

### Statistical analysis

Data analysis was performed using SPSS 22.0 software (IBM Corporation, Armonk, NY, USA). The count data were analyzed using Chi squared and Fisher’s exact probability tests, whereas the measurement data were analyzed using *t*-test. Survival curves were analyzed using the Kaplan–Meier and log-rank tests, and relationships between ANXA8 expression and clinicopathological parameters were analyzed using the Cox regression model. *P* < 0.05 indicated statistically significant differences.

## Results

### Bioinformatics analyses revealed differential mRNA expression levels of annexins in ovarian cancer

**Fig. 1:**
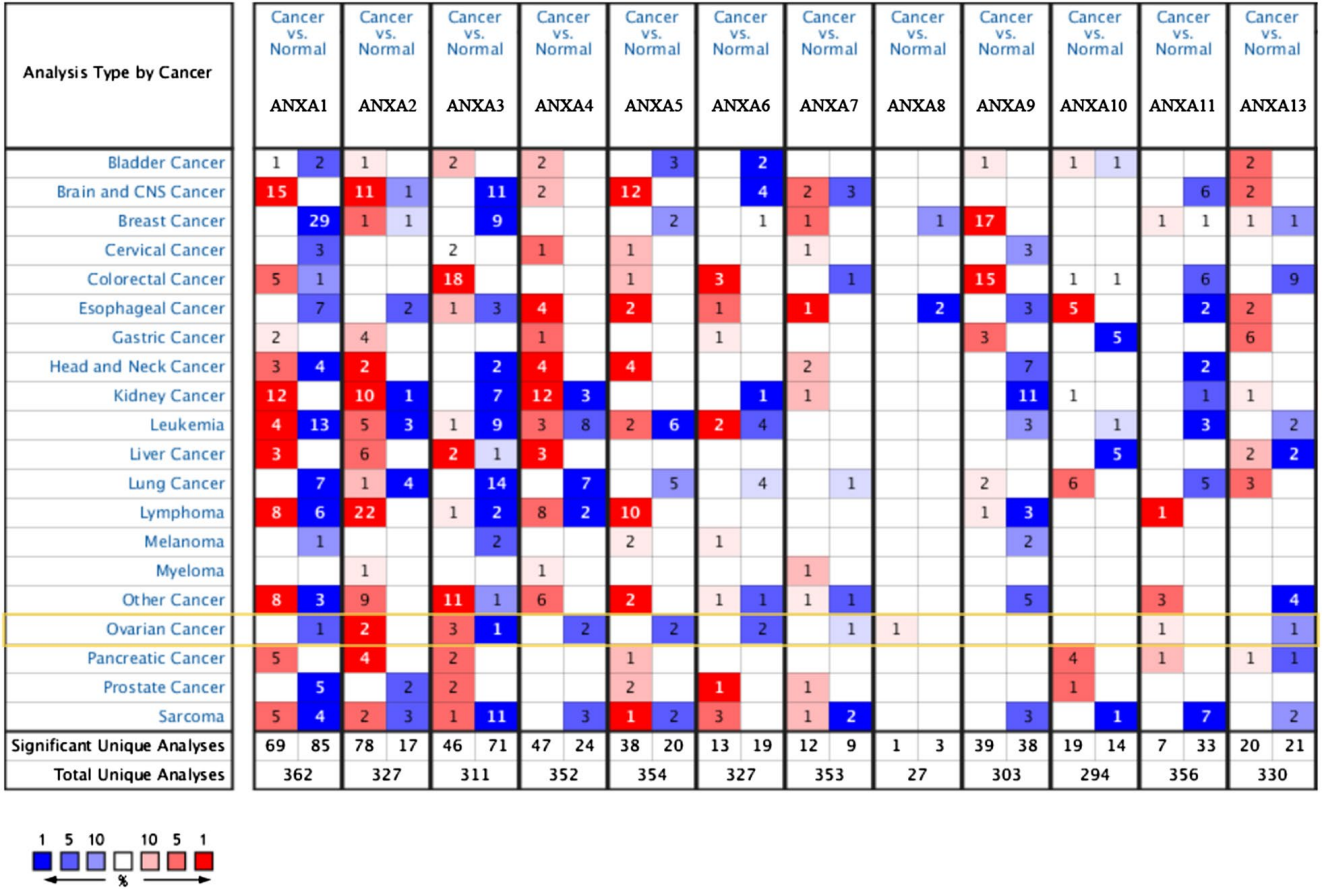
mRNA expression levels of Annexins in various types of cancer (Oncomine). The threshold was designed with following parameters: fold change = 2 and *P*-value = 0.01. The cell number represents the number of dataset that meets the thresholds. The color intensity (red or blue) is directly proportional to the significance level of upregulation or downregulation, respectively. mRNA expression levels of Annexins in ovarian cancer are delineated with yellow highlights

The mRNA expression levels of Annexins in different types of cancer were analyzed using the Oncomine database (Fig. 1 and Table 1). Welsh et al. compared the mRNA expression levels between 28 cases of ovarian serous surface papillary carcinoma and 4 cases of normal ovarian tissues, and found that *ANXA2* expression was significantly upregulated in malignant tumor tissues (*P* = 3.11E−8, fold change = 2.003) [23]. Similar results were reported by Lu et al. in ovarian mucinous adenocarcinoma (*P* = 7.94E−6, fold change = 2.411) [24]. Welsh et al., Yoshihara et al. and Adib et al. reported that *ANXA3* was highly expressed in ovarian serous carcinoma (*P* = 3.65E−5, fold change = 17.581; *P* = 2.50E−4, fold change = 2.121; *P* = 0.002; fold change = 5.145) [25, 26]. However, the study carried out by Bonome et al. showed that *ANXA3* was downregulated based on 185 cases of ovarian carcinoma (*P* = 1.55E−12, fold change = − 7.270) [22]. Yoshihara et al. compared mRNA expression levels of 43 cases of ovarian serous adenocarcinoma and 10 cases of peritoneum, and found that *ANXA8* expression was significantly upregulated in malignant tumor tissues (*P* = 6.08E−4, fold change = 5.452) [25]. Welsh et al. also found that *ANXA11* was upregulated in ovarian serous surface papillary carcinoma (*P* = 1.79E−4, fold change = 2.140) [23]. In addition, the expression levels of *ANXA1*, *ANXA4*, *ANXA5*, *ANXA6*, *ANXA7* and *ANXA13* were lower in ovarian cancer than in the normal controls. Bonome et al. also found that *ANXA1* (*P* = 3.82E−9, fold change = − 2.654), *ANXA4* (*P* = 2.64E−8, fold change = − 3.067), and *ANXA5* (*P* = 4.43E−7, fold change = − 2.849) were downregulated in ovarian cancer [22]. The TCGA database confirmed that *ANXA4* expression was downregulated in ovarian serous cystadenocarcinoma (*P* = 4.81E−6, fold change = − 2.019) and that *ANXA13* was downregulated in malignant tumor tissues (*P* = 1.68E−4, fold change = − 9.347). Yoshihara et al. found that *ANXA5* (*P* = 1.77E−9, fold change = − 3.973) and *ANXA6* (*P* = 4.86E−14, fold change = − 5.732) were downregulated in ovarian serous adenocarcinoma compared with the peritoneum [25], which was confirmed by Bonome et al. and Welsh et al. [22, 23]. Welsh et al. found that *ANXA7* was downregulated in malignant tumor tissues (*P* = 0.006, fold change = − 2.217) [23]. Relatively few studies have been conducted for other members of the Annexin family in the Oncomine database.

**Table 1:** Datasets of Annexins in ovarian cancer (Oncomine)

Gene	Tumor (cases)	Normal (cases)	Fold change	t-test	*P*-value	Dataset
ANXA1	Ovarian carcinoma (185)	Ovarian surface epithelium (10)	− 2.654	− 9.268	3.82E−9	Bonome et al.
ANXA2	Ovarian serous surface papillary carcinoma (28)	Ovary (4)	2.003	8.097	3.11E−8	Welsh et al.
	Ovarian mucinous adenocarcinoma (9)	Ovarian surface epithelium (5)	2.411	6.988	7.94E−6	Lu et al.
ANXA3	Ovarian serous surface papillary carcinoma (28)	Ovary (4)	17.581	12.161	3.65E−5	Welsh et al.
	Ovarian serous adenocarcinoma (43)	Peritoneum (10)	2.121	3.863	2.50E−4	Yoshihara et al.
	Ovarian serous adenocarcinoma (6)	Ovary (4)	5.145	4.731	0.002	Adib et al.
	Ovarian carcinoma (185)	Ovarian surface epithelium (10)	− 7.270	− 18.366	1.55E−12	Bonome et al.
ANXA4	Ovarian carcinoma (185)	Ovarian surface epithelium (10)	− 3.067	− 10.005	2.64E−8	Bonome et al.
	Ovarian serous cystadenocarcinoma (586)	Ovary (8)	− 2.019	− 9.354	4.81E−6	TCGA
ANXA5	Ovarian serous adenocarcinoma (43)	Peritoneum (10)	− 3.973	− 10.436	1.77E−9	Yoshihara et al.
	Ovarian carcinoma (185)	Ovarian surface epithelium (10)	− 2.849	− 8.941	4.43E−7	Bonome et al.
ANXA6	Ovarian serous adenocarcinoma (43)	Peritoneum (10)	− 5.732	− 12.820	4.86E−14	Yoshihara et al.
	Ovarian serous surface papillary carcinoma (28)	Ovary (4)	− 2.043	− 8.881	2.12E−6	Welsh et al.
ANXA7	Ovarian serous surface papillary carcinoma (28)	Ovary (4)	− 2.217	− 2.667	0.006	Welsh et al.
ANXA8	Ovarian serous adenocarcinoma (43)	Peritoneum (10)	5.452	4.001	6.08E−4	Yoshihara et al.
ANXA11	Ovarian serous surface papillary carcinoma (28)	Ovary (4)	2.140	4.862	1.79E−4	Welsh et al.
ANXA13	Ovarian serous cystadenocarcinoma (586)	Ovary (8)	− 9.347	− 6.439	1.68E−4	TCGA

*TCGA* The Cancer Genome Atlas

**Fig. 2:**
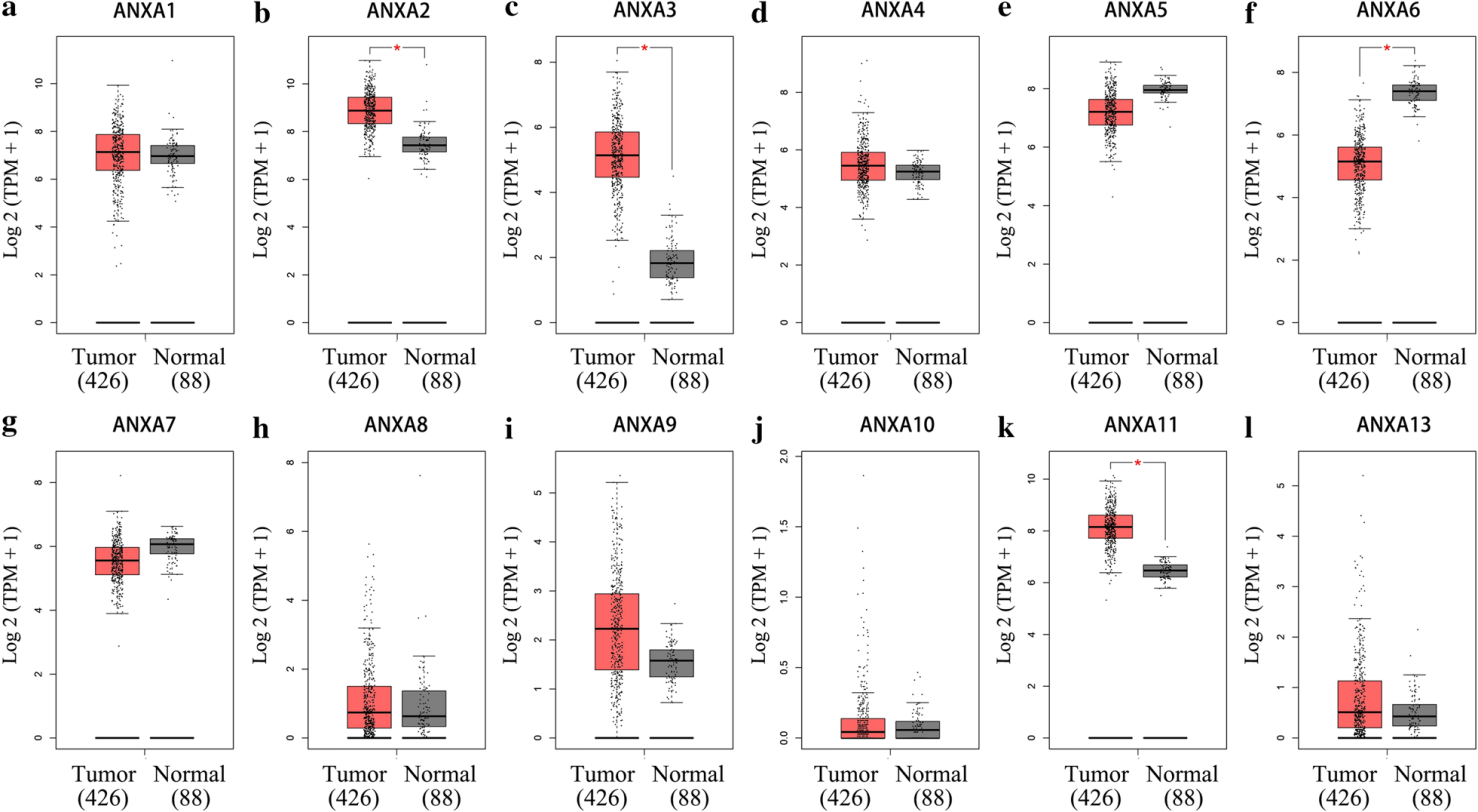
mRNA expression levels of Annexins in ovarian cancer and normal ovarian tissues (GEPIA). **a**–**l** mRNA expression levels of each member of the Annexin family in ovarian cancer and normal ovarian tissues. Box plots show mRNA expression of Annexins in ovarian tumor (red plot) and the corresponding normal tissues (gray plot). Axis units are Log2 (TPM + 1). *P < 0.01

In addition, we analyzed the differential mRNA expression levels of Annexins between ovarian cancer and normal ovarian tissues using the GEPIA database (Fig. 2), which indicated that *ANXA2*, *ANXA3*, and *ANXA11* mRNA expression levels were significantly upregulated in ovarian cancer (Fig. 2b, c, k). In contrast, *ANXA6* expression was significantly downregulated (Fig. 2f). The results are generally consistent with those obtained using the Oncomine database. Both databases showed that *ANXA2*, *ANXA3*, *ANXA8*, and *ANXA11* mRNA expression levels were upregulated in ovarian cancer compared with normal tissues, while *ANXA5*,*ANXA6*, and *ANXA7* mRNA expression levels were downregulated. In addition, although there were no differences in mRNA expression levels of *ANXA1*, *ANXA4*, and *ANXA13* between ovarian cancer and normal tissues in the GEPIA database, downregulation of these genes was found in the Oncomine database.

**Fig. 3:**
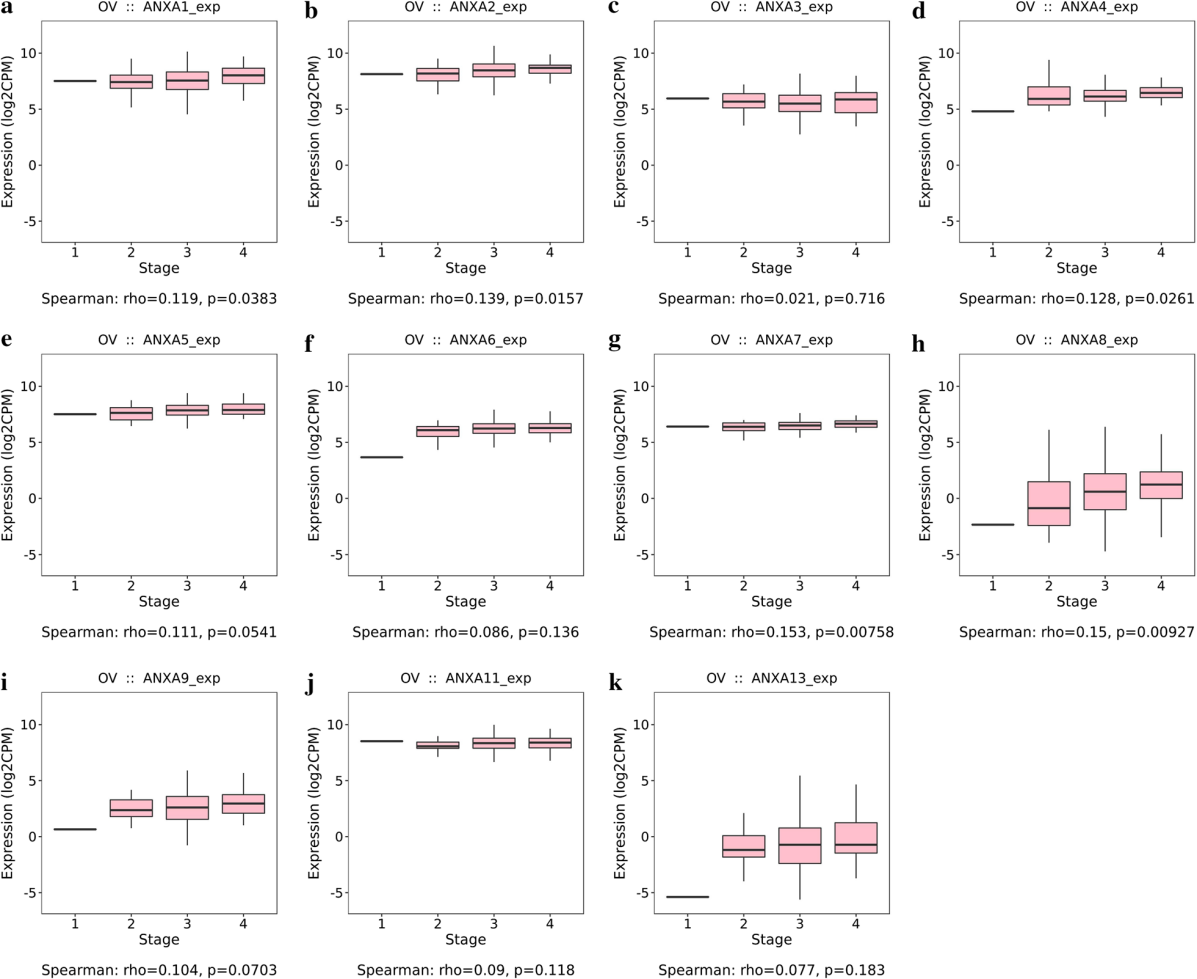
Correlation analysis of the Annexins expression and clinical stages in ovarian serous adenocarcinoma (TISIDB). **a**–**k** Correlation between the expression of each Annexin family member and clinical stages in ovarian serous adenocarcinoma. Spearman’s correlation analysis between biomarkers and clinical stages is based on whole clinical stages. No study was available regarding the ANXA10 expression in ovarian serous adenocarcinoma

Further analysis using the TISIDB database to study the relationship between Annexin mRNA expression levels and the whole clinical stages of ovarian serous adenocarcinoma (Fig. 3) showed that the expression levels of *ANXA1* (Fig. 3a, Spearman: ρ = 0.119, *P* = 0.0383), *ANXA2* (Fig. 3b, Spearman: ρ = 0.139, *P* = 0.0157), *ANXA4* (Fig. 3d, Spearman: ρ = 0.128, *P* = 0.0261), *ANXA7* (Fig. 3g, Spearman: ρ = 0.153, *P* = 0.00758) and *ANXA8* (Fig. 3h, Spearman: ρ = 0.15, *P* = 0.00927) increased significantly with advanced FIGO stages. However, the expression levels of *ANXA3* (Fig. 3c), *ANXA5* (Fig. 3e), *ANXA6* (Fig. 3f), *ANXA9* (Fig. 3i), *ANXA11* (Fig. 3j), and *ANXA13* (Fig. 3k) did not change significantly with FIGO stage. Combining the results of three databases, *ANXA2* and *ANXA8* mRNA expression levels were upregulated in ovarian cancer and the expression levels increased significantly with advanced FIGO stages.

### Amplification, deletion, mutation, and fusion of Annexin genes in ovarian cancer

**Fig. 4:**
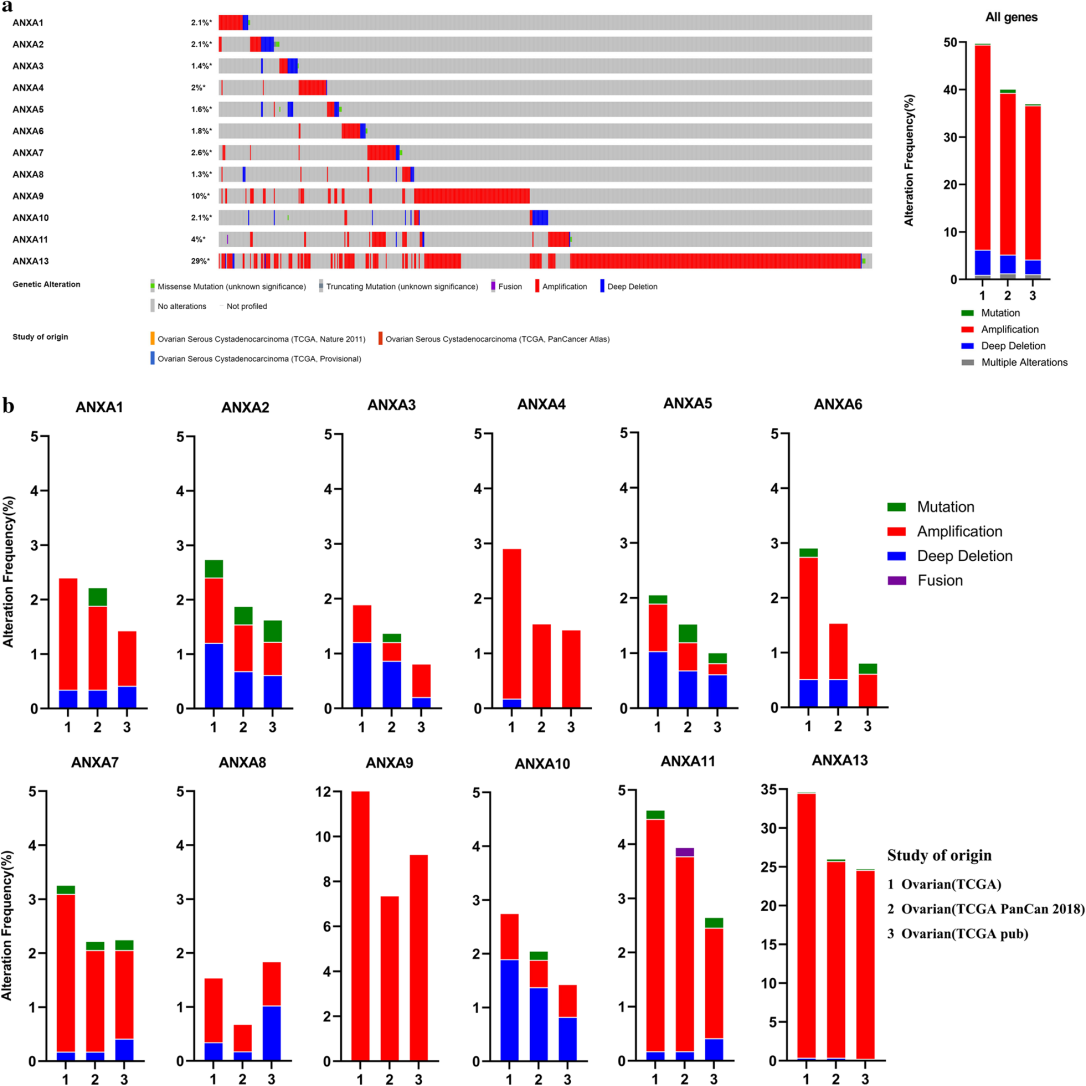
Analyses of genetic variations in Annexin genes in ovarian cancer (cBioPortal). **a** OncoPrint visual summary of variations on a query of Annexin family members and overview of the analyses of genetic variations in Annexin genes. **b** Analyses of genetic variations in Annexin family members reported in different studies. The variations frequency included amplification (red), deep deletions (blue), mutation (green), fusion (purple) and multiple alterations (grey). *TCGA* The Cancer Genome Atlas

Genetic variations of Annexins in 1680 cases retrieved from three studies (489 cases from TCGA, Nature 2011; 585 cases from TCGA, PanCancer Atlas; and 606 cases from TCGA, Provisional) were analyzed using the cBioPortal database (Fig. 4). We found varying degrees of genetic variation among the 12 Annexin family members, among which *ANXA13* displayed the highest incidence rate (34.65% in TCGA) of genetic variations (the incidence rates of amplification, deep deletion, and mutation were 34.13%, 0.34%, and 0.17%, respectively), followed by *ANXA9* whose incidence rate of amplification was 12.01% (in TCGA). Most genetic variations in Annexins were amplifications, although *ANXA3* (whose incidence rates of deep deletion and amplification were 1.20% and 0.69%, respectively), *ANXA5* (whose incidence rates of deep deletion, amplification, and mutation were 1.03%, 0.86%, and 0.17%, respectively), and *ANXA10* (whose incidence rates of deep deletion and amplification were 1.89% and 0.86%, respectively) had higher probabilities of deletion events. Amplifications, deep deletions, and mutations were found in *ANXA1*, *ANXA2*, *ANXA3*, *ANXA5*, *ANXA6*, *ANXA7*, *ANXA10*, and *ANXA13*. In addition, *ANXA11* had gene fusion events (0.17% in TCGA PanCan).

### ANXA8 showed the greatest prognostic value in patients with ovarian serous tumors

**Fig. 5:**
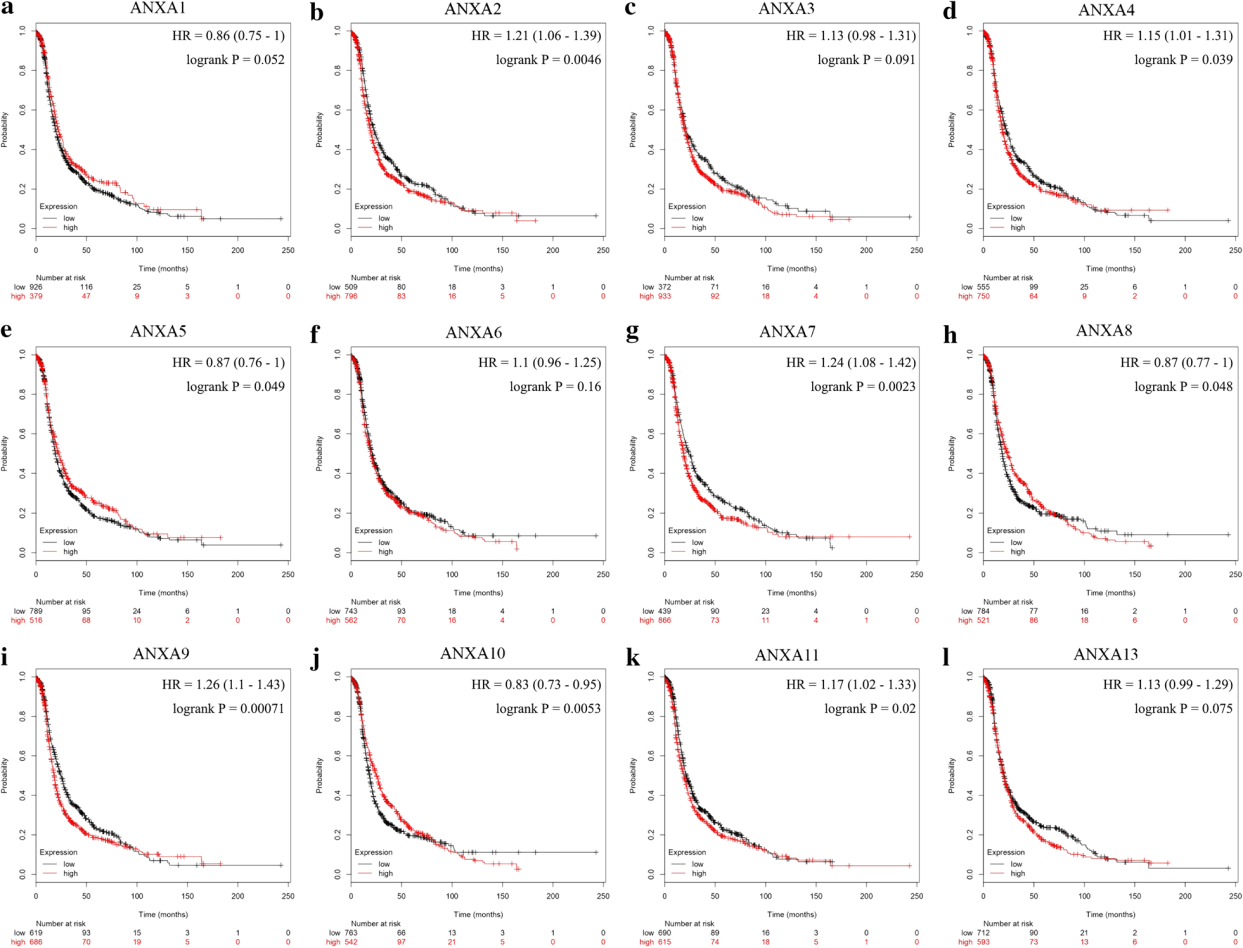
Prognostic values of Annexins in ovarian cancer (PFS in Kaplan–Meier plotter). **a**–**l** Prognostic significance of individual Annexin members in ovarian cancer. The *P*-values were calculated using the log-rank test. *PFS* progression-free survival

Correlations between the mRNA expression levels of Annexins and PFS in patients with ovarian cancer were analyzed using the Kaplan–Meier plotter database (Fig. 5). The analysis revealed that the mRNA expression levels of *ANXA2* (Fig. 5b), *ANXA4* (Fig. 5d), *ANXA5* (Fig. 5e), *ANXA7* (Fig. 5g), *ANXA8* (Fig. 5h), *ANXA9* (Fig. 5i), *ANXA10* (Fig. 5j), and *ANXA11* (Fig. 5k) were correlated with patient prognosis. Hence, Annexins are of great significance for assessing the prognosis of ovarian cancer.

**Fig. 6:**
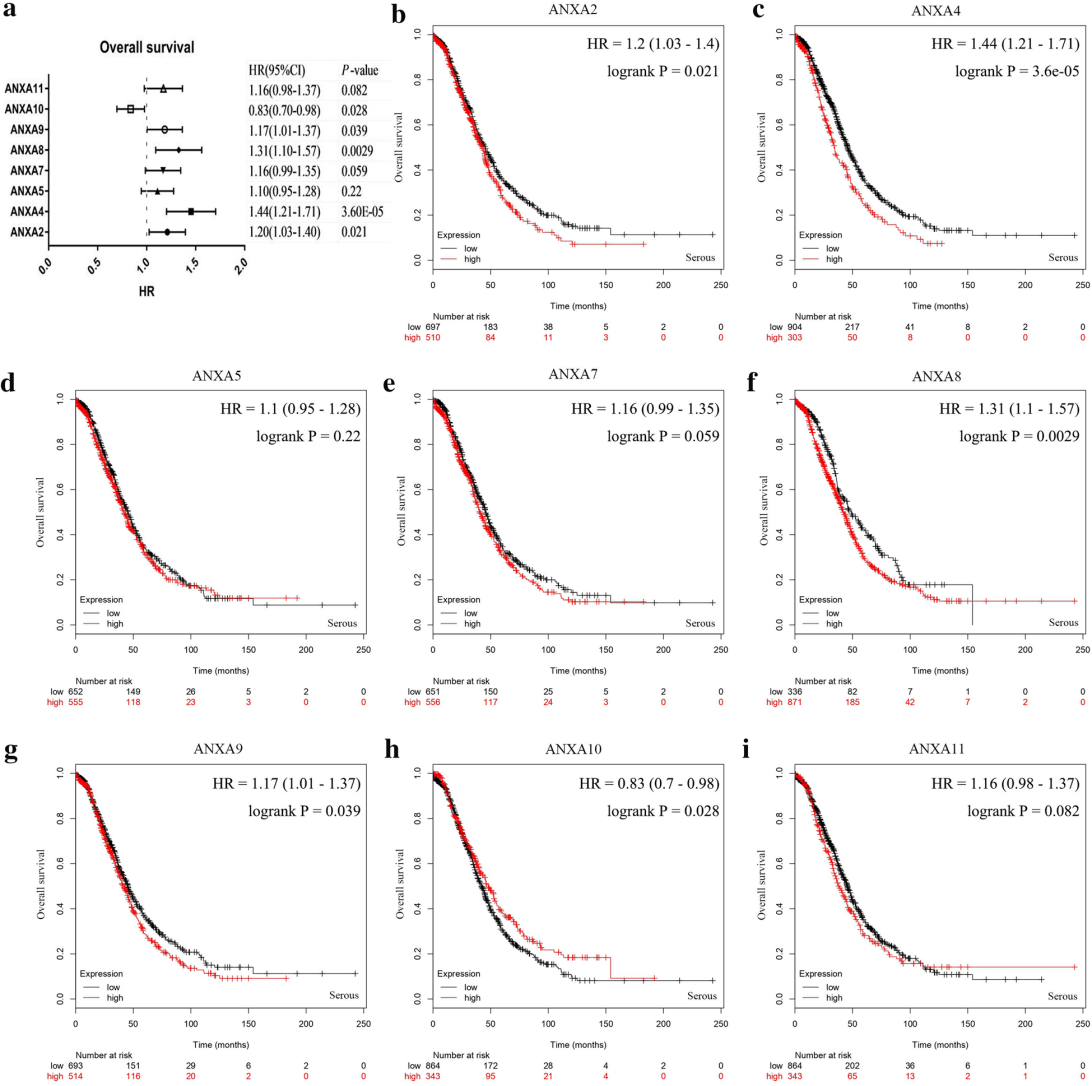
Prognostic values of Annexins in ovarian serous tumors (OS in Kaplan–Meier plotter). **a** Prognostic HR of individual Annexin family members in ovarian serous tumor. **b**–**i** Prognostic significance of individual Annexin members in ovarian serous tumor. The *P*-values were calculated using the log-rank test. *OS* overall survival

**Fig. 7:**
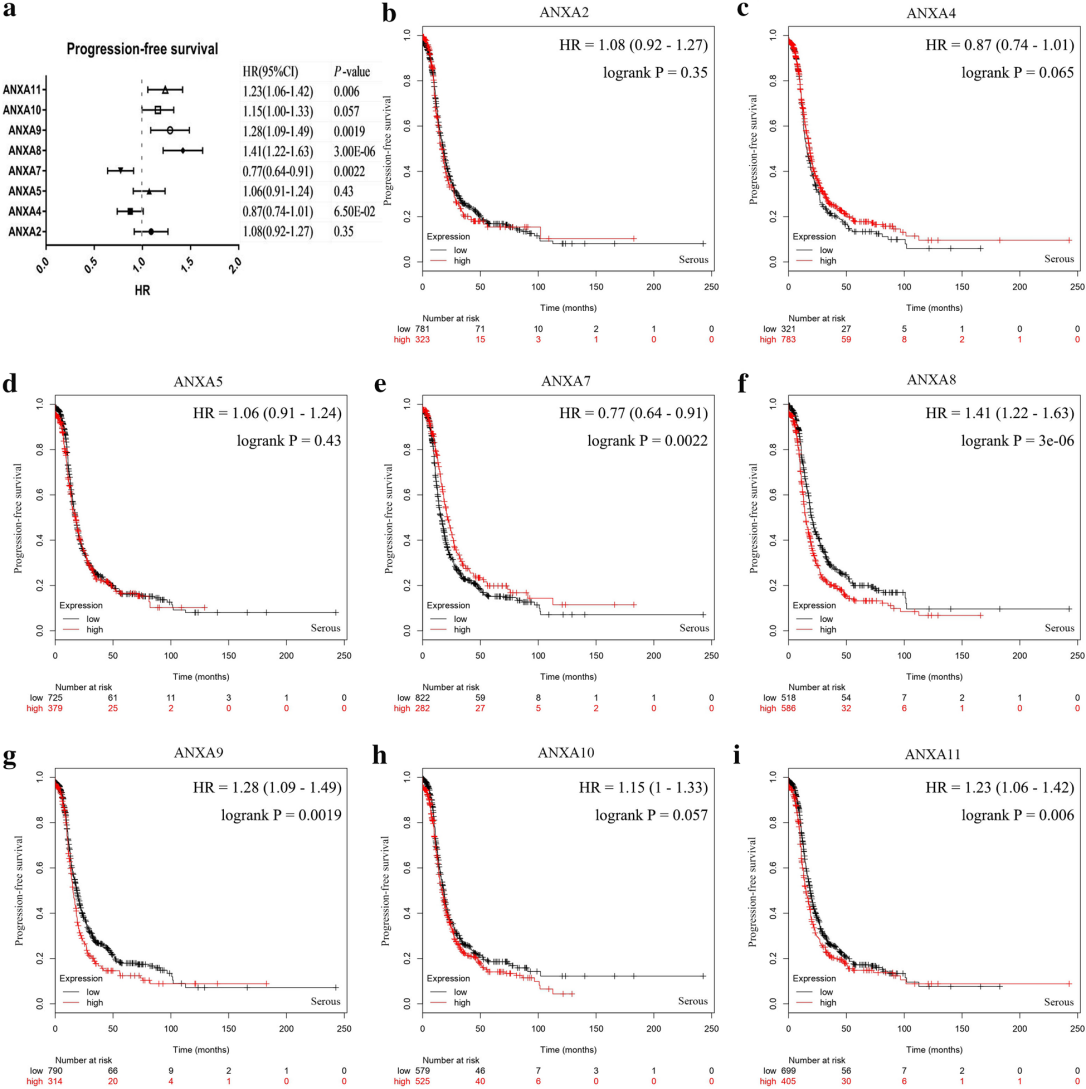
Prognostic values of Annexins in ovarian serous tumors (PFS in Kaplan–Meier plotter). **a** Prognostic HRs of individual Annexin members in ovarian serous tumors. **b**–**i** Prognostic significance of individual Annexin members in ovarian serous tumors. The *P*-values were calculated using the log-rank test. *PFS* progression-free survival

Ovarian serous tumors are the most common pathological type of ovarian tumors. Therefore, the expression levels of those PFS-associated genes in ovarian cancer were compared to analyze their correlations with overall survival (OS) and PFS in ovarian serous tumors (Figs. 6, 7). Upregulation of *ANXA2* (Fig. 6b), *ANXA4* (Fig. 6c), *ANXA8* (Fig. 6f), and *ANXA9* (Fig. 6g) mRNAs displayed significant correlations with poor OS in patients with ovarian serous tumors. In contrast, *ANXA10* (Fig. 6h) mRNA upregulation signified a better prognosis, which was consistent with its correlation with the PFS of all types of ovarian cancer. Figure 7 shows that upregulation of *ANXA8* (Fig. 7f), *ANXA9* (Fig. 7g), and *ANXA11* (Fig. 7i) mRNAs was significantly correlated with poor PFS in patients with ovarian serous tumors, whereas *ANXA7* (Fig. 7e) mRNA downregulation was correlated with poor PFS. Taken together, these data indicated that the upregulation of *ANXA8* and *ANXA9* mRNAs was significantly correlated with poor OS and PFS in patients with ovarian serous tumors.

**Fig. 8:**
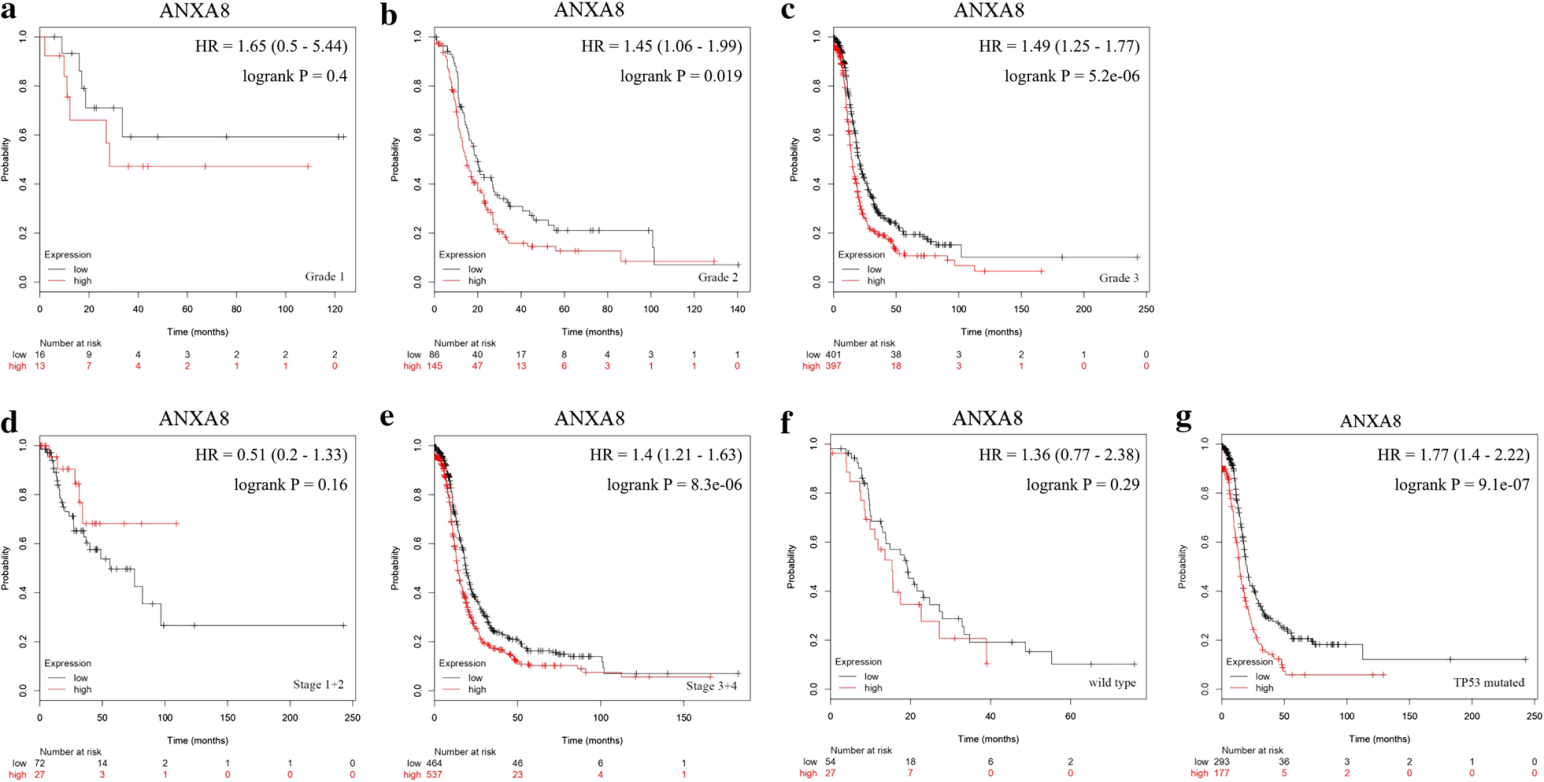
Survival analyses of *ANXA8* in ovarian serous tumors (PFS in Kaplan–Meier plotter). **a**–**c** Prognostic significance of *ANXA8* expression in ovarian serous tumor with different grades. **d**–**e** Prognostic significance of *ANXA8* in ovarian serous tumor with different FIGO stages. **f**–**g** Prognostic significance of *ANXA8* in ovarian serous tumors with or without *TP53* mutations. The *P*-values were calculated using the log-rank test. *PFS* progression-free survival, *FIGO* International Federation of Gynecology and Obstetrics

*ANXA8*, which showed the greatest prognostic significance for OS and PFS in patients with ovarian serous tumors, was selected to study correlations between its mRNA expression level and PFS during different degrees of differentiation, FIGO stages, and *TP53* mutations (Fig. 8). *ANXA8* mRNA upregulation was correlated with poor PFS with moderate and poor differentiation, and the correlation was even more pronounced with poor differentiation (Fig. 8b, c). *ANXA8* mRNA upregulation indicated poor PFS in patients with FIGO stage III–IV (Fig. 8e). A significant correlation was observed between of *ANXA8* mRNA upregulation and poor PFS in patients with *TP53* mutations, when compared to patients with wild-type *TP53* (Fig. 8g). Therefore, *ANXA8* can better reflect the prognosis in patients with ovarian serous tumors, and its correlation with prognosis was even more pronounced in patients with poor differentiation, advanced FIGO stages, and *TP53* mutations.

### Correlations between Annexin family members and construction of a gene–gene interaction network

**Fig. 9:**
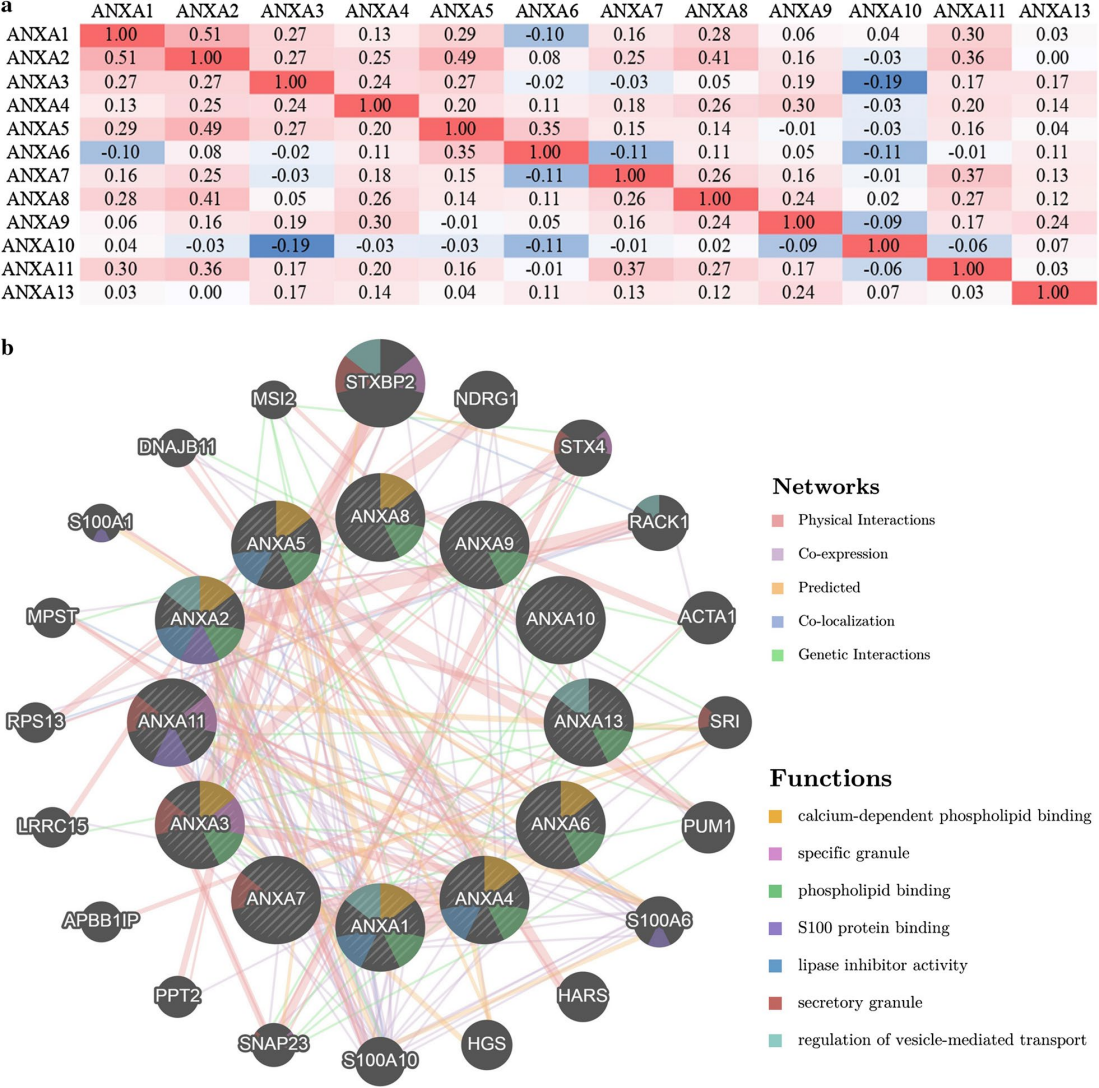
Correlation analysis and gene–gene interaction network of Annexin family members (cBioPortal and GeneMANIA). **a** Spearman’s correlation of Annexin family members. Red and blue cells indicate positive and negative correlations, respectively. The color intensity is directly proportional to the strength of the correlations. **b** Gene–gene interaction network among Annexin family members. Each node represents a gene. The node size represents the strength of interactions. The inter-node connection lines represent the types of gene–gene interactions, and the line color represents the types of interactions. The node color represents the possible functions of respective genes

The Spearman’s correlations between Annexin family members in ovarian cancer were determined by online analyses using the cBioPortal database (TCGA, PanCancer Atlas; Fig. 9a). Spearman’s correlation coefficient exceeding 0.30 indicated a good correlation. The results indicated a positive correlation among *ANXA2* with *ANXA1* (*r* = 0.51, *P* = 1.07E−14), *ANXA5* (*r* = 0.49, *P* = 1.07E−13), *ANXA8* (*r* = 0.41, *P* = 9.86E−10) and *ANXA11* (*r* = 0.36, *P* = 1.07E−07); *ANXA5* with *ANXA6* (*r* = 0.35, *P* = 3.90E−07); and *ANXA7* with *ANXA11* (*r* = 0.37, *P* = 9.54E−08) (Additional file 1).

A gene–gene interaction network for the 12 Annexin genes was constructed, and their functions were analyzed using the GeneMANIA database (Fig. 9b). The 12 central nodes representing Annexin family members were surrounded by 20 nodes representing genes that greatly correlated with the family in terms of physical interactions, co-expression, predictions, co-localization, and genetic interactions. The top five genes displaying the greatest correlations with the Annexin family included *STXBP2* (syntaxin binding protein 2), *NDRG1* (N-myc downstream regulated 1), *STX4* (syntaxin 4), *RACK1* (receptor for activated C kinase 1), and *ACTA*1 (actin alpha 1, skeletal muscle), among which *STXBP2* was correlated with *ANXA3* and *ANXA11* in terms of physical interactions, and co-localized with *ANXA3*. *NDRG1* was correlated with *ANXA5* in terms of physical interactions and was co-expressed with *ANXA3*, *ANXA4*, and *ANXA6*. *STX4* was correlated with *ANXA3* in terms of physical interactions and was co-expressed with *ANXA2*, *ANXA5*, and *ANXA11*. *RACK1* was correlated with *ANXA2* in terms of physical interactions. In addition, *ACTA1* was correlated with *ANXA1* and *ANXA8* in terms of physical interactions and correlated with *ANXA5* in terms of genetic interactions (Additional file 2). Further functional analysis revealed that these proteins showed the greatest correlation with calcium-dependent phospholipid binding (FDR = 5.80e−12). Additionally, these proteins were correlated with specific granules, phospholipid binding, S100 protein binding, lipase inhibitor activity, secretory granules, regulation of vesicle-mediated transport, post-Golgi vesicle-mediated transport, calcium-dependent protein binding, and enzyme-inhibitor activity (Additional file 3).

### Functional and pathway enrichment analyses of ANXA8

**Fig. 10:**
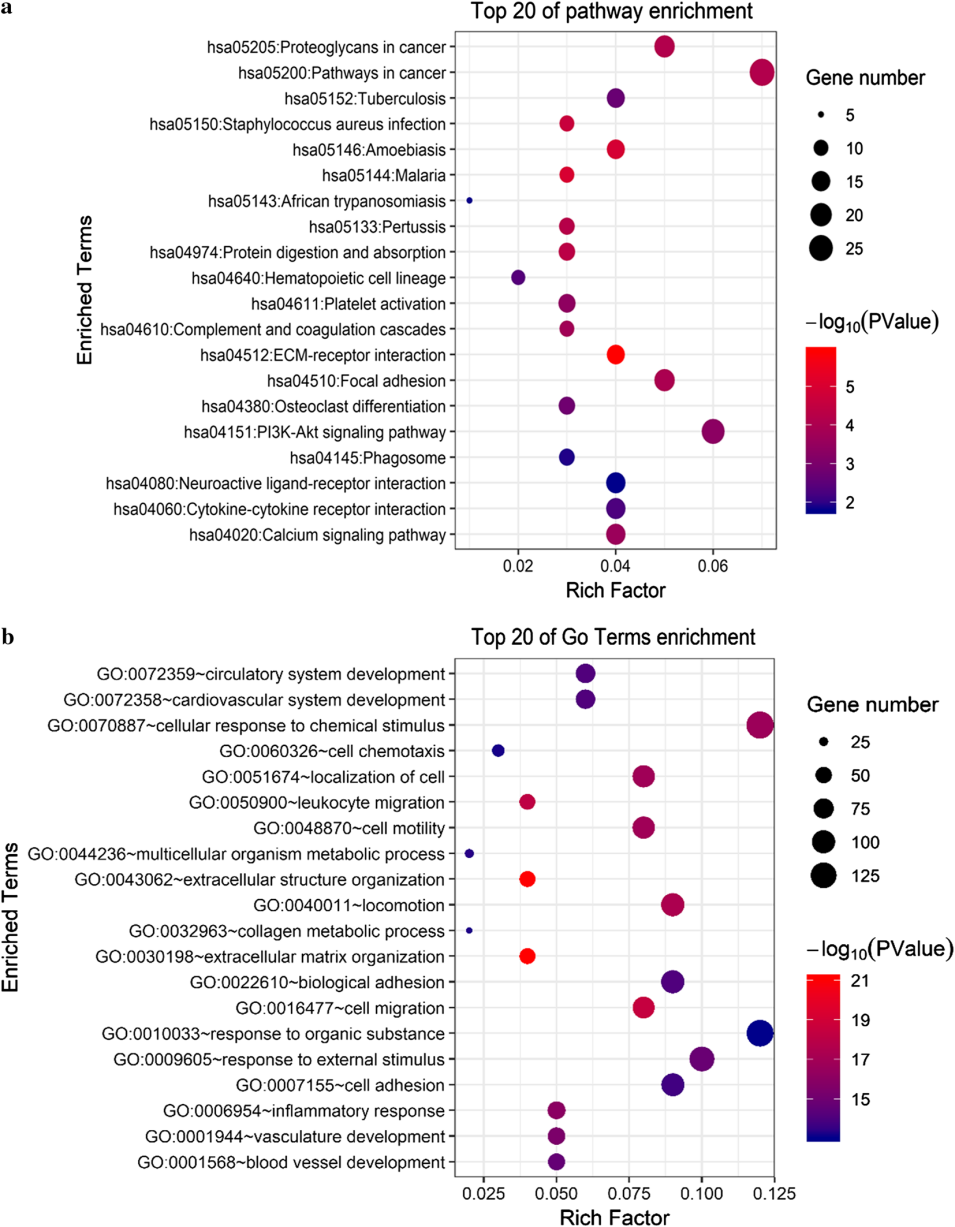
Functional and pathway enrichment analyses of genes co-expressed with ANXA8 (DAVID). **a** Bubble plot representing enriched pathways of the genes co-expressed with ANXA8. **b** Bubble plot representing the enriched functions of genes co-expressed with ANXA8. Y-axis: name of the signaling pathway or function; X-axis: percentage of the number of genes assigned to a term among the total number of genes annotated in the network; Bubble size: number of genes assigned to a pathway or function; Color: enriched *P*-value; Red bubble: indicates a greater significance level. *GO* gene ontology

A total of 491 genes co-expressed with *ANXA8* having an average Spearman’s correlation coefficient of 0.35 were obtained from the cBioPortal database based on the selection criteria (Additional file 4). The genes co-expressed with *ANXA8* were subjected to functional and pathway enrichment analyses using the DAVID database (version 6.8). Bubble plots representing the top 20 enriched biological functions and pathways (based on *P*-value) were constructed using the *ggplot2.R* package (Fig. 10). Among signaling pathways showing the greatest correlation for genes co-expressed with *ANXA8*, hsa04151 (PI3K-Akt signaling pathway), hsa05205 (proteoglycans in cancer), hsa05200 (pathways in cancer), hsa04510 (focal adhesion), hsa04512 (ECM-receptor interaction), and hsa04020 (calcium signaling pathway) were correlated with the tumorigenesis and progression of tumors (Fig. 10a). The functional analysis results showed that the following biological processes were enriched for the genes co-expressed with *ANXA8*: GO:0048870 (cell motility), GO:0022610 (biological adhesion), GO:0016477 (cell migration), GO:0007155 (cell adhesion), GO:0006954 (inflammatory response), and GO:0001568 (blood vessel development), as shown in Fig. 10b (Additional files 5, 6).

### Regulation of immune molecules by ANXA8

**Fig. 11:**
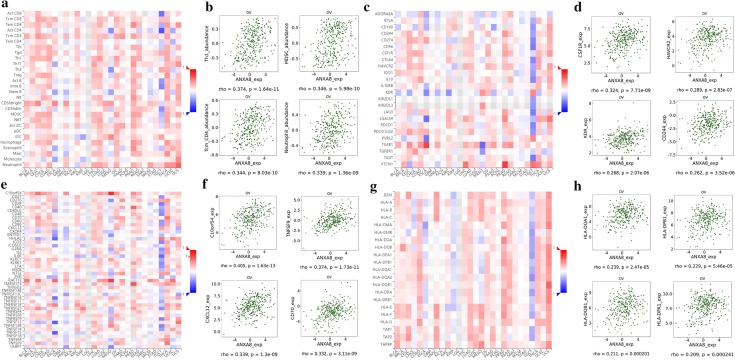
Spearman’s correlation of *ANXA8* with lymphocytes and immunomodulators (TISIDB). **a** Relations between the abundance of TILs and *ANXA8* expression. **b** Top 4 TILs displaying the greatest Spearman’s correlation with *ANXA8* expression. **c** Relations between the abundances of immunoinhibitors and *ANXA8* expression. **d** Top 4 immunoinhibitors displaying the greatest Spearman’s correlation with *ANXA8* expression. **e** Relations between abundances of immunostimulators and *ANXA8* expression. **f** Top 4 immunostimulators displaying the greatest Spearman’s correlation with *ANXA8* expression. **g** Relations between abundance of MHC molecules and *ANXA8* expression. **h** Top 4 MHC molecules displaying the greatest Spearman’s correlation with *ANXA8* expression. Red and blue cells indicate positive and negative correlations, respectively. The color intensity is directly proportional to the strength of the correlations. *TILs* tumor-infiltrating lymphocytes, *MHC* major histocompatibility complex

The Spearman’s correlations between *ANXA8* expression and lymphocytes and immunomodulators were analyzed using the TISIDB database (Fig. 11). Figure 11a shows the correlation between *ANXA8* expression and tumor-infiltrating lymphocytes (TILs), and the lymphocytes displaying the greatest correlations included type-1 T helper cell (Th1; Spearman: ρ = 0.374, *P* = 1.64e−11), myeloid-derived suppressor cells (MDSCs; Spearman: ρ = 0.346, *P* = 5.98e−10), central memory CD4 T cells (Tcm_CD4; Spearman: ρ = 0.344, *P* = 8.03e−10), and neutrophils (Spearman: ρ = 0.339, *P* = 1.36e−09) (Fig. 11b). Immunomodulators can be further classified into immunoinhibitors, immunostimulators, and major histocompatibility complex (MHC) molecules. Figure 11c shows correlations between *ANXA8* expression levels and immunoinhibitors. The immunoinhibitors displaying the greatest correlations included CSF1R (Spearman: ρ = 0.324, *P* = 7.71e−09), HAVCR2 (Spearman: ρ = 0.289, *P* = 2.83e−07), KDR (Spearman: ρ = 0.268, *P* = 2.07e−06), and CD244 (Spearman: ρ = 0.262, *P* = 3.52e−06) (Fig. 11d). Figure 11e shows correlations between *ANXA8* expression and immunostimulators, and the immunostimulators displaying the greatest correlations included C10orf54 (Spearman: ρ = 0.405, *P* = 1.63e−13), TNFSF9 (Spearman: ρ = 0.374, *P* = 1.73e−11), CXCL12 (Spearman: ρ = 0.339, *P* = 1.3e−09), and CD70 (Spearman: ρ = 0.332, *P* = 3.11e−09) (Fig. 11f). Figure 11g shows correlations between *ANXA8* expression and MHC molecules, and the MHC molecules displaying the greatest correlations included HLA-DQA1 (Spearman: ρ = 0.239, *P* = 2.47e−05), HLA-DPB1 (Spearman: ρ = 0.229, *P* = 5.46e−05), HLA-DQB1 (Spearman: ρ = 0.211, *P* = 0.000201), and HLA-DPA1 (Spearman: ρ = 0.209, *P* = 0.000241) (Fig. 11h). Therefore, *ANXA8* may be involved regulating the above immune molecules.

### IHC validation of high ANXA8 expression in epithelial ovarian cancer

**Fig. 12:**
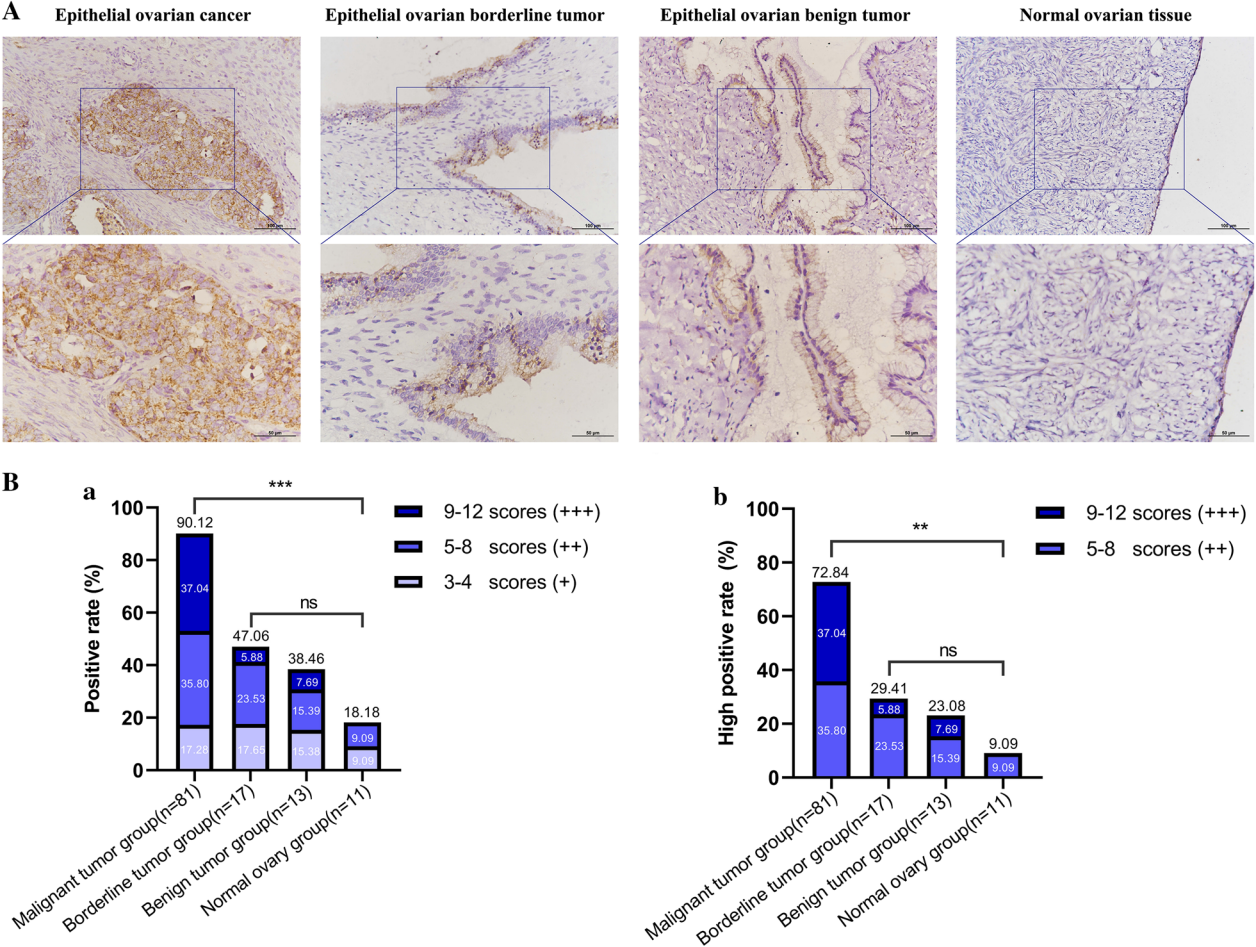
Expression levels of ANXA8 in ovarian tissues from different groups. **A** Representative images of immunohistochemical staining in epithelial ovarian cancer, epithelial ovarian borderline tumor, epithelial ovarian benign tumor and normal ovarian tissue. Scale bars: upper, 100 μm; lower, 50 μm. The lower figure is a partial enlargement of the blue box above. **B** Statistical analysis of ANXA8 expression in ovarian tissues from different groups. **a** Comparison of positive rates of ANXA8 expression among the different groups. **b** Comparison of high positive rates of ANXA8 expression among the different groups. Chi square test, **P < 0.01, ***P < 0.001

IHC assays demonstrated that ANXA8 was mainly localized on the cell membrane and in the cytoplasm. ANXA8 positive rate and high positive rate in the malignant tumor group (90.12% and 72.84%, respectively) were significantly higher than those in the borderline tumor group (47.06% and 29.41%, respectively), benign tumor group (38.46% and 23.08%, respectively), and normal ovary group (18.18% and 9.09%, respectively; *P* < 0.05). No significant pairwise differences were observed in the positive rate and high positive rate between the borderline tumor group, benign tumor group, and normal ovary group (*P* > 0.05, as shown in Fig. 12).

### ANXA8 expression in epithelial ovarian cancer was associated with the FIGO stage

Eighty-one patients with epithelial ovarian cancer were divided into high ANXA8-expression groups (++/+++) and low ANXA8-expression groups (−/+). Statistical analyses revealed a significant correlation between ANXA8 expression and FIGO stages (*P* = 0.006), where patients at FIGO stage III–IV had significantly higher ANXA8 high positive rate (84.78%) than patients at FIGO stage I–II (57.14%). ANXA8 showed the highest high positive rate (87.50%) in clear cell carcinoma and the lowest high positive rate in mucinous cystadenocarcinoma (55.56%). However, the ANXA8 expression level did not significantly correlate with the degree of differentiation, lymph node metastasis, and pathologic type (*P* > 0.05), as shown in Table 2.

**Table 2:** Relationships between ANXA8 expression in epithelial ovarian cancer and clinicopathological parameters

Characteristics	n	Low		High		High positive rate (%)	*P*-value
		(−)	(+)	(++)	(+++)		
FIGO stage							0.006*
I–II	35	6	9	8	12	57.14	
III–IV	46	2	5	21	18	84.78	
Differentiation							0.401
Well–moderate	38	3	9	13	13	68.42	
Poor	43	5	5	16	17	76.74	
LN metastasis							0.414
No	45	6	8	15	16	68.89	
Yes	19	0	4	8	7	78.95	
No lymphadenectomy	17	2	2	6	7	76.47	
Pathologic type							0.673
Serous	40	3	8	15	14	72.50	
Mucinous	9	1	3	3	2	55.56	
Endometrioid	11	2	1	3	5	72.72	
Clear cell carcinoma	8	0	1	2	5	87.50	
Poorly differentiated adenocarcinoma	13	2	1	6	4	76.92	

*FIGO* International Federation of Gynecology and Obstetrics, *LN* lymph node

* *P* < 0.05

### High ANXA8 expression was an independent risk factor affecting the survival and prognosis in patients with epithelial ovarian cancer

**Fig. 13:**
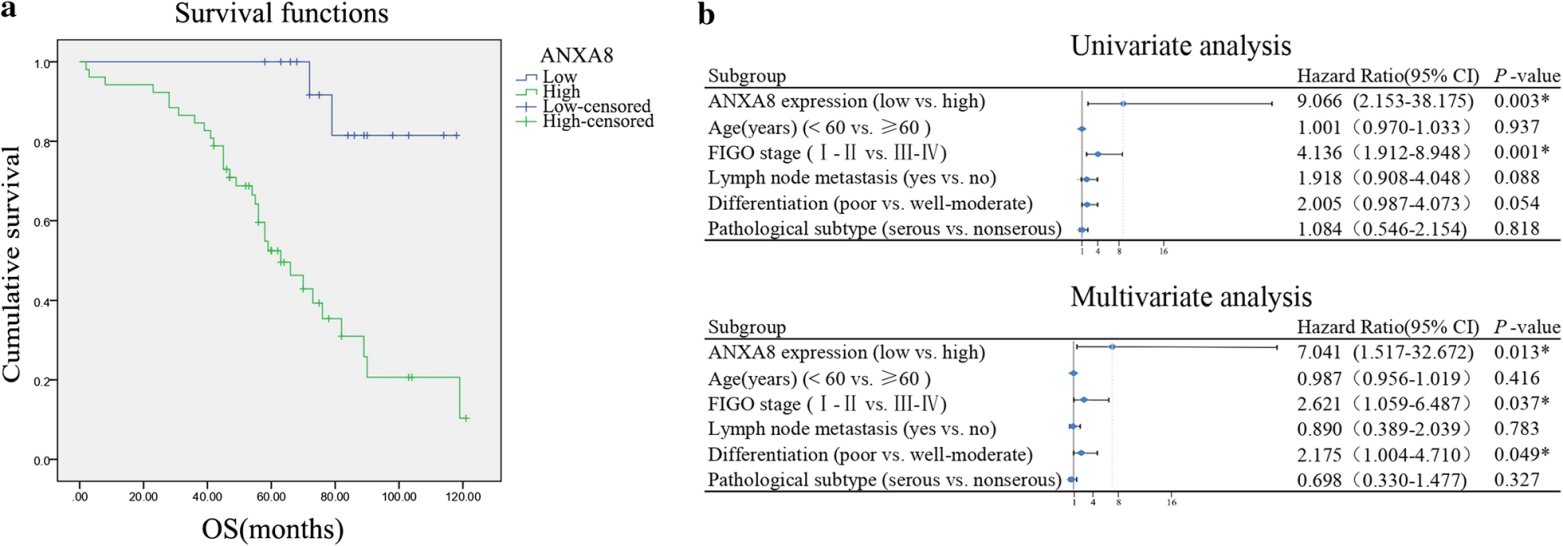
Correlations of ANXA8 expression with the survival and prognosis of patients with epithelial ovarian cancer. **a** Relationship between ANXA8 expression and the OS of patients with epithelial ovarian cancer. **b** Forest map based on univariate and multivariate Cox regression analysis of the OS of patients with epithelial ovarian cancer. *P < 0.05. *OS* overall survival, *CI* confidence interval, *FIGO* International Federation of Gynecology and Obstetrics

The 81 patients with epithelial ovarian cancer were followed up (the last patient follow-up was carried out on September 30, 2017), among which valid information was available for 52 patients (64.20%), while 29 patients (35.80%) who were lost to follow-up. The OS of patients who were lost to follow-up was defined as the period from the date of surgery to the date of the last follow-up. Analyses using Kaplan–Meier and log-rank tests revealed that patients in the high ANXA8-expression group had significantly lower 5-year survival rates than patients in the low ANXA8-expression group (*P* < 0.01; Fig. 13a). A Cox regression model was adopted to analyze relationships between the survival times of ovarian cancer patients with ANXA8 expression, age, clinical stage, lymph node metastasis, degree of differentiation, and the pathological type. The results of univariate analysis suggested that significant correlations occurred between ANXA8 expression levels (*P* = 0.003), FIGO stages (*P* = 0.001), and the OS of patients. Multivariate analysis indicated that the high ANXA8 expression (*P* = 0.013), late FIGO stages (*P* = 0.037), and poor differentiation (*P* = 0.049) were independent risk factors affecting the survival and prognosis of patients with epithelial ovarian cancer (Fig. 13b).

## Discussion

Annexins belong to a multigene superfamily of Ca^2+^-regulated, phospholipid-binding proteins that are dysregulated in various malignant tumors and are involved in various biological processes, such as signal transduction, proliferation, and apoptosis of tumor cells. However, the roles of Annexin family members in malignant tumors, especially ovarian cancer, have rarely been studied, and their mechanisms of function have yet to be elucidated. In this study, we investigated the expression, prognostic value, genetic variations, and potential roles of Annexin family members in ovarian cancer for the first time. ANXA8, which displayed the greatest correlation with the prognosis of patients with ovarian cancer, as well as the closest association with its tumorigenesis and progression, was selected for subsequent in-depth investigations of its biological processes and signaling pathways, as well as its correlations with immunity. We also validated the expression of ANXA8 in ovarian cancer by performing IHC assays and analyzed relationships between its expression with clinicopathological parameters, survival, and prognosis in patients with ovarian cancer.

Our research group aimed to study correlations between Annexin family members and ovarian cancer. The results suggested that ANXA2 and ANXA4 were correlated with ovarian cancer tumorigenesis and progression. We confirmed that ANXA2 was highly expressed in ovarian cancer, especially serous and mucinous cystadenocarcinoma, and that its high expression was closely associated with lymph node metastasis. ANXA2 and human epididymis protein 4 (HE4) are interacting proteins, and the binding between them can activate various signaling pathways, such as the MAPK and FOCAL pathways, thereby promoting the invasion, metastasis, and epithelial-mesenchymal transition (EMT) of ovarian cancer cells [27]. Our results also showed that ANXA4 was highly expressed in ovarian cancer, and in particular, it displayed the highest expression level in ovarian clear cell carcinoma. Binding between ANXA4 and Vimentin promotes the proliferation, invasion, and migration of ovarian clear cell carcinoma, and inhibits cell apoptosis by activating the ERK and NF-kB pathways. Both ANXA2 and ANXA4 are Lewis y-modified proteins whose biological functions are further enhanced by glycosylation [28, 29]. The above results are consistent with the results reported by Mogami et al. [30]. In addition, Madureira PA et al. illustrated that two molecules of ANXA2 bind to a dimer of the protein S100A10 to form a heterotetramer, also known as AIIt. The ANXA2 subunit helps to stabilize and anchor S100A10 to the plasma membrane, and AIIt activates plasminogen via tissue-type plasminogen activator (t-PA) and urokinase-type plasminogen activator (uPA). This in turn increases the production of plasmin, leading to the activation of metalloproteinases (MMPs) and the degradation of extracellular matrix (ECM) proteins, thereby promoting tumor progression and chemoresistance [31, 32]. Liu et al. found that ovarian cancer proliferation and invasion can be suppressed by inhibiting ANXA2 via β-catenin/EMT [33]. Cua et al. presented evidence revealing that the monoclonal antibody 2448 can be used to monitor the EMT of ovarian and breast cancers by targeting a unique glycan epitope on ANXA2 [34]. These findings complemented our preliminary studies. Both the Oncomine and GEPIA database analyses showed that *ANXA2* mRNA expression level was significantly upregulated in ovarian cancer. The TCGA database mainly focused on ANXA4 expression level in ovarian serous cystadenocarcinoma, which may have led to a discrepancy with our results that showed the highest expression level of ANXA4 in ovarian clear cell carcinoma. Prognostic analysis suggested that *ANXA2* and *ANXA4* mRNA upregulation was significantly correlated with poor OS in patients with ovarian serous tumors. Therefore, we believe that ANXA2 and ANXA4 are closely associated with the tumorigenesis and progression of ovarian cancer.

In addition, our preliminary prediction based on microarray data suggested that *ANXA8* may be closely associated with ovarian cancer tumorigenesis and progression. Screening for differentially expressed genes in three groups of ovarian cancer cell lines with different degrees of malignancy showed that *ANXA8* was highly expressed in cell lines with greater degrees of malignancy and drug resistance, and its expression pattern was consistent with that of α1,2-fucosyltransferase [14]. It was recently confirmed that ANXA8 induced HIF-1α transcription via calcium signaling pathways in pancreatic cancer, and can be regulated by zinc-finger transcription factor ZIC2 to inhibit apoptosis [13, 35]. The bioinformatics prediction performed by Rossetti et al. suggested that all-trans retinoic acid receptor alpha participates in the morphogenesis of breast cancer by directly targeting the promoter region of *ANXA8* or targeting the 3′-untranslated region of ANXA8 mRNA via miR-342 [12]. However, no report has demonstrated a correlation between ANXA8 expression and ovarian cancer.

The correlation between ANXA8 expression and ovarian cancer was further validated using various databases. The Oncomine database showed that *ANXA8* mRNA expression level was significantly upregulated in ovarian cancer, and the TISIDB database showed that *ANXA8* mRNA expression increased significantly with advanced FIGO stages. The results of the prognostic analysis showed that, among Annexin family members, the upregulation of *ANXA8* mRNA expression displayed the greatest correlation with poor OS and PFS in patients with ovarian serous tumors. In addition, *ANXA8* showed an even more significant correlation with prognosis in patients with poor differentiation, advanced FIGO stages, and *TP53* mutations. Hence, we speculate that *ANXA8* is extremely important for evaluating the survival and prognosis in patients with ovarian cancer and may serve as an indicator for *TP53* mutations in patients with ovarian cancer.

The genes co-expressed with *ANXA8* identified using the cBioPortal database were subjected to functional and pathway enrichment analyses, and the results indicated that they are mainly involved in biological processes, such as cell migration, cell adhesion, angiogenesis, and inflammatory responses, as well as in regulating various cancer related signaling pathways, such as PI3K-Akt, focal adhesion, and proteoglycans in cancer, thereby affecting the tumorigenesis and progression of ovarian cancer. It has been reported that ANXA8 affects the formation of CD63/VEGFR2/β1 integrin complexes and promotes the VEGF-A-mediated human umbilical vein endothelial cell sprouting [36]. EGF-mediated FOXO4 phosphorylation in cholangiocarcinoma leads to the transcriptional inhibition of *ANXA8*, FAK downregulation, and alteration of F-actin kinetics [11]. Therefore, we infer that *ANXA8* and its co-expressed genes jointly regulate ovarian cancer tumorigenesis and progression through a complex regulatory network.

In recent years, increasing attention has been paid to the treatment of tumors via immunotherapy, which targets cancer by activating the body’s own immune system. Recent findings have suggested that ovarian cancer is an immunogenic tumor and, thus, developing vaccines, adoptive T cell therapy, and immunomodulators has broad prospects for reducing mortality rates in patients with ovarian cancer [37]. In this study, we assessed the correlation between *ANXA8* and the immune system via the TISIDB database, and the results showed that *ANXA8* had the greatest correlation with lymphocytes (such as Th1, MDSC, Tcm_CD4, Neutrophil), immunoinhibitors (such as CSF1R, HAVCR2, KDR, and CD244), immunostimulators (such as C10orf54, TNFSF9, CXCL12, and CD70), and MHC molecules (such as HLA-DQA1, HLA-DPB1, HLA-DQB1, and HLA-DPA1). The epigenetic silencing of Th1-type chemokines is a novel mechanism of immune evasion in tumors, and selective epigenetic reprogramming can enhance the clinical efficacy of treatments against ovarian cancer [38]. As the most notable biomarker in tumor research in the past decade,membrane-bound PD-L1 in ovarian cancer cells can be induced by soluble inflammatory factors derived from tumor associated macrophage (TAM), thus leading to immune escape [39]. Dual blockade of CXCL12-CXCR4 and PD-1-PD-L1 pathways have been found to inhibit ovarian tumor growth and prevent immunosuppression [40]. In addition, osteopontin overexpression upregulates PD-L1 expression in hepatocellular carcinoma cells by activating the CSF1–CSF1R pathway in macrophages, and blockage of CSF1/CSF1R prevents TAM trafficking. Hence, CSF1R inhibitors can be used to synergistically enhance the therapeutic efficacy of PD-L1 antibodies [41]. Therefore, *ANXA8*, which is associated with these immune molecules, may provide a new target for studying the immune evasion of ovarian cancer cells and can potentially serve as a immunotherapeutic target for ovarian cancer.

IHC assays demonstrated that ANXA8 was found mainly in the cell membrane and cytoplasm, and was highly expressed in epithelial ovarian cancer. In addition, its expression level was correlated with FIGO stages. Patients with high ANXA8 expression and advanced FIGO stages had a relatively poor prognosis. COX regression model analysis suggested that high ANXA8 expression was an independent risk factor affecting the survival and prognosis in patients with epithelial ovarian cancer. Therefore, ANXA8 has the greatest correlation with prognosis in patients with ovarian cancer and is extremely closely related to ovarian cancer tumorigenesis and progression. ANXA8 is a high candidate as a biomarker for early diagnosis, immunotherapy, and prognostic judgments of ovarian cancer.

In addition, correlations between other members of the Annexin family and ovarian cancer were validated using these databases. The results indicated that they were also correlated with ovarian cancer tumorigenesis and progression.

Previous studies confirmed that some members of the Annexin family are associated with chemoresistance in ovarian cancer. ANXA3 was upregulated in platinum-resistant ovarian cancer [7], whereas ANXA1 and ANXA11 expression was negatively correlated with paclitaxel and cisplatin resistance in ovarian cancer, respectively [42, 43]. The Oncomine database showed that *ANXA1* mRNA expression was downregulated in ovarian cancer, while both of the databases showed that *ANXA3* and *ANXA11* mRNA expression levels were significantly upregulated in ovarian cancer, among which the upregulation of *ANXA11* mRNA displayed a significant correlation with poor PFS in patients with ovarian serous tumors.

There are relatively few studies on other members of the Annexin family in ovarian cancer, but it has been demonstrated that they are dysregulated in other malignant tumors, such as breast cancer, gastric cancer, colorectal cancer, and pancreatic cancer [44–49]. The results of our database analyses showed that the mRNA expression levels of *ANXA5, ANXA6*, *ANXA7* and *ANXA13* were downregulated. *ANXA7* mRNA downregulation was significantly correlated with poor PFS in patients with ovarian serous tumors, and *ANXA9* mRNA upregulation was significantly correlated with poor OS and PFS in patients with ovarian serous tumors.

Cancer cells carry different types of mutations that can lead to uncontrolled cell replication. Copy number alterations are ubiquitous in cancer, and are associated with cancer outcomes such as recurrence and death [50]. Although some studies have suggested that copy number variations correlate with mRNA expression [51], we found that gene amplification of Annexin family and high mRNA expression were not significantly associated. However, we found gene alteration in 49.74% of Annexin family in the TCGA database, including an amplification rate of 43.22%, and a deep deletion rate of 5.32%. The incidence rate of genetic variations in the *ANXA13* gene was up to 34.65%, whereas the incidence rate of amplifications in the *ANXA9* gene was up to 12.01%. Therefore, genetic variations in *ANXA9* and *ANXA13* genes may be correlated with ovarian cancer tumorigenesis and progression. In addition, *ANXA2*, *ANXA4*, *ANXA8*, *ANXA9*, and *ANXA11*, for which mRNA upregulation were significantly associated with poor OS or PFS, had a higher incidence rate of amplifications than deep deletions. Therefore, increased amplifications of Annexin family may be associated with poor prognosis in ovarian cancer.

To clarify the mechanisms of function of Annexins in ovarian cancer, we constructed a gene–gene interaction network using the GeneMANIA database. The results suggested that Annexins interact intensively with other genes, such as *STXBP2*, *NDRG1*, *STX4*, *RACK1*, and *ACTA1*. It has been demonstrated that *NDRG1* interacts with the Wnt co-receptor, LRP6, to inhibit the EMT mediated by the Wnt-β-catenin signaling pathway [52]. The interaction between STX4 and Muncl18c facilitates invadopodium formation in tumor cells [53]. RACK1 promotes the phosphorylation of Akt and MAPK, as well as the proliferation, migration, and invasion of ovarian cancer cells [54]. It has also been confirmed that RACK1 and ANXA7 are interacting proteins that participate in liver cancer metastasis [55]. Therefore, these interacting genes are closely associated with the tumorigenesis and progression of tumors, but their interactions with Annexins in ovarian cancer still require further experimental validations. Our functional analyses indicated that Annexins were correlated with calcium-dependent phospholipid binding and S100 protein binding. Jaiswal et al. demonstrated that S100A11 and ANXA2 form a complex at the site of injury which facilitates local remodeling of the actin cytoskeleton and excision of damaged cell membrane [56]. Hatoum et al. identified differential regulation of the annexin/S100A family by activation of p14ARF-p53-p21 in breast cancer cells, and demonstrated that ANXA1, ANXA2, ANXA4, ANXA6, and ANXA9 were upregulated, with a high expression of ANXA9 functioning as a predictor for poor OS after endocrine therapy in estrogen receptor-positive (ER+) patients [57].

Therefore, Annexins may be closely associated with and play important roles in ovarian cancer. However, functional mechanisms of Annexins still require further experimental validations, owing to differences found among databases, limited sample sizes, and few relevant experimental studies.

## Conclusions

In summary, Annexin family members display varying degrees of abnormal expressions, and play important roles in the tumorigenesis and progression of ovarian cancer. Our data revealed that ANXA8 was significantly highly expressed in malignant ovarian tumor tissues and that high ANXA8 expression was significantly correlated with poor prognosis in patients with ovarian cancer. Therefore, ANXA8 may prove to be a novel biomarker for the early diagnosis, immunotherapy, and prognostic judgment of patients with ovarian cancer.

## Supplementary information

**Additional file 1.** Spearman’s correlation of Annexin family members (cBioPortal).

**Additional file 2.** Construction of gene–gene interaction network for Annexin family members (GeneMANIA).

**Additional file 3.** Related function of gene–gene interaction network for Annexin family members (GeneMANIA).

**Additional file 4.** A total of 491 co-expressed genes of ANXA8 (cBioPortal).

**Additional file 5.** Pathway enrichment analysis of ANXA8 (DAVID).

**Additional file 6.** Gene Ontology analysis of ANXA8 (DAVID).

## Data Availability

Not applicable.

## Abbreviations

ANXA8: Annexin A8
IHC assay: immunohistochemical assay
GEPIA: Gene Expression Profiling Interactive Analysis
DAVID: the Database for Annotation, Visualization, and Integrated Discovery
OS: overall survival
PFS: progression-free survival
GSEA: gene set enrichment analysis
TCGA database: The Cancer Genome Atlas database
HR: hazard ratio
CI: confidence interval
FIGO: International Federation of Obstetrics and Gynecology
OC: ovarian cancer
TIL: tumor-infiltrating lymphocytes
MHC: major histocompatibility complex
LN: lymph node
TAM: tumor associated macrophage
